# Extracorporeal Shock Wave Therapy Promotes Osteogenic Differentiation in a Rabbit Osteoporosis Model

**DOI:** 10.3389/fendo.2021.627718

**Published:** 2021-03-25

**Authors:** Baofeng Li, Renkai Wang, Xianyin Huang, Yongliang Ou, Zhenyu Jia, Shanghui Lin, Ying Zhang, Hong Xia, Bei Chen

**Affiliations:** ^1^Department of Radiation Oncology, Nanfang Hospital, Southern Medical University, Guangzhou, China; ^2^Department of Orthopaedics, General Hospital of Southern Theater Command of PLA, Guangdong Key Lab of Orthopedic Technology and Implant Materials, Guangzhou, China; ^3^School of Clinical Medicine,Guangdong Pharmaceutical University, Guangzhou, China

**Keywords:** extracorporeal shock wave, osteoblast, osteoporosis, osteogenic differentiation, osteoporosis model, SMAD2

## Abstract

Extracorporeal shock wave therapy (ESWT) has been identified to accelerate bone formation. However, detailed mechanism has not been fully explained. In this study, we found that ESWT promoted osteoblast formation *in vitro*. Local ESW treatment of femur increased bone formation *in vivo*. Furthermore, changing the density or frequency of energy, there was no statistical difference in osteogenic differentiation. Therapeutically, local ESW therapy relieved bone loss and increased the number of bone trabecular in a rabbit osteoporosis model and promoted endogenous levels of SMAD2 protein expression. Thus, ESWT may be a potential therapy by promoting osteoblast maturation through TGF-β/SMAD2 pathway.

## Introduction

Osteoporosis is a major chronic skeletal disease characterized by decreased bone mass and deterioration of bone microarchitecture, finally resulting in fragility fracture. With the aging of population, the incidence of osteoporosis is increasingly serious, bringing heavy medical and socioeconomic burden to countries.

Extracorporeal shock wave (ESW), a short duration acoustic wave that carry energy and can propagate through soft tissues, is a cost-effective and non-invasive modality, which is widely used in clinic for the treatment of various diseases, mainly including renal calculus, erectile dysfunction, and musculoskeletal disorder (1–3). It is believed that Extracorporeal shock wave therapy (ESWT) has positive effects on wound healing, angiogenesis, tissue regeneration, and bone remodeling *via* physical interaction (4, 5). A growing number of studies demonstrated that ESWT has shown promising outcomes in the treatment of fracture, osteonecrosis, osteoarthritis, as well as osteoporosis (3, 6, 7). However, the therapeutic effect of ESWT on bone-loss diseases and its underlying mechanism are still indistinct.

In this study, we generated osteoporosis rabbit model to evaluate the protective efficiency of ESWT on bone loss. Bone mesenchymal stem cells (BMSC) were cultured *in vitro* and treated with ESWT to investigate the potential mechanism whereby ESWT promoted bone formation. This study aimed to illustrate the therapeutic effect and the molecular mechanism of ESWT on protecting against bone loss in osteoporosis rabbit model, which may provide theoretical basis for clinical application of ESWT on the treatment of bone-loss diseases.

## Materials and Methods

### Cell Culture *In Vitro*

We obtained bone marrow from femurs from 8-month-old female New Zealand white rabbits and then centrifuged it at 1,000 rpm for 10 min. The supernatant was discarded and BMSCs were washed with DMEM medium containing 15% fetal bovine serum and cultured at 37°C in a 5% CO2 humidified incubator. To induce osteogenic differentiation, BMSCs were seeded in 12-well plates and a commercial kit was used to differentiate the cells into osteoblast.

For ESW therapy, we seeded BMSC or RAW264.7 cells (1 × 10^4^) into each well of 12-well plates and performed ESW with the shock wave sensor facing upwards. Then, we set the pulse frequency to 4 Hz and the focal length (penetration depth) 0.5 cm. The energy density and impulse times were adjusted as previously described.

### Alizarin Red Staining

We seeded BMSC cells (1 × 10^4^) into each well of 12-well plates and cultured them in α-MEM (Gibco, USA) supplemented with 10% FBS (Gibco, USA) and 1% penicillin–streptomycin (Gibco, USA). To induce osteogenic differentiation, BMSCs were seeded in 12-well plates and a commercial kit was used to differentiate the cells into osteoblast for 21 days. Then, we discarded the culture medium, the cells were washed with PBS and fixed with 95% alcohol for 10–30 min, and stained with 1% Alizarin Red for 10 min to analyze the activity of osteoblast mineralization.

### Alkaline Phosphatase (ALP) Staining

We seeded BMSC cells (1 × 10^4^) into each well of 24-well plates and cultured them in α-MEM (Gibco, USA) supplemented with 10% FBS (Gibco, USA) and 1% penicillin–streptomycin (Gibco, USA). To induce osteogenic differentiation, BMSCs were seeded in 12-well plates and a commercial kit was used to differentiate the cells into osteoblast for 7 days. After washing with PBS, the cells were fixed with cold acetone for 10 min, rinsed with distilled water, incubated in the incubation solution at 37°C for 4 h, rinsed with tap water, soaked in 2% cobalt nitrate for 3–5 min, and then washed with distilled water, rinsed with 1% ammonium sulfide for 2 min, rinse with tap water after sealing with gum.

### Osteoclastogenesis Assays

RAW264.7 cells were seeded on 24-well plates (1 × 10^4^) in D-MEM (Gibco, USA) supplemented with 10% FBS (Gibco, USA) and 1% penicillin–streptomycin (Gibco, USA). We used M-CSF (30 ng/ml) and RANKL (100 ng/ml) to promote osteoclast maturation for 7 days. Then, we performed TARP-staining according to general protocols. TRAP positive cells (more than five nucleus) were considered as mature osteoclasts.

### Real-Time PCR

We isolated total RNA from BMSCs by TRIzol reagent (Life Technologies, USA) and synthesized complementary DNAs (cDNAs) from total RNA. Then, we performed quantitative reverse transcriptase PCR (qRT-PCR) by FastStart Universal SYBR Premix ExTaqTM II (Takara Biotechnology, Japan) on an ABI Prism 7900 HT Sequence detection system (Applied Biosystems, USA). We used 100 μl RNA in RT-PCR experiments. The reaction conditions were as follows: 95°C, 30 s; 95°C, 10 s; 60–65°C, 10 s; for 40 cycles. The primer sequences used for qRT-PCR were as follows: Alp: forward, 5′-CCA ACT CTT TTG TGC CAG AGA-3′ and reverse, 5′-GGC TAC ATT GGT GTT GAG CTT TT-3′; Ocn: forward, 5′- CTG CGC TCT GTC TCT CTG AC-3′ and reverse, 5′- TTA AGC TCA CAC TGC TCC CG-3′; OPG: forward, 5′- AGT GTG AGG AAG GGC GTT AC-3′ and reverse, 5′-AAT GTG CTG CAG TTC GTG TG-3′; Runx2: forward, 5′-AAA TGC CTC CGC TGT TAT GAA-3′ and reverse, 5′-GCT CCG GCC CAC AAA TCT-3′.

### Animal Models and ESWT

All animal experiments were conducted in accordance with the National Institute of Health (NIH) Guide for the Care and Use of Laboratory Animals, with the approval of the Animal Care and Use Committee of Fourth Military Medical University (No. 2019110801). Female New Zealand white rabbits (8 months old) were obtained from the Animal Center of Fourth Military Medical University. The rabbits were housed in barrier housing conditions in the Animal Center of Fourth Military Medical University. Gengerate a rabbit osteoporosis model, we used the protocol as described previously (8). Briefly, we fasted the rabbits for 24 h prior to surgery. The OVX group received bilateral OVX through a ventral incision under general anesthesia with intramuscular injection of pentobarbital sodium (50 mg/kg). The negative control group received sham surgery. Then, we fasted all the animals for another 12 h, and administered antibiotic prophylaxis with cefonicid sodium (50 mg/kg) before and during the 3 days following surgery. Rabbits in the negative control and OVX groups were injected with 0.9% benzyl alcohol (50 mg/kg).

For ESW therapy, the shock wave treatment machine (Epos Ultra, Dornier MedTech, Wessling, Germany) was used for ESW treatment. This shock wave generator was an electromagnetic pulse type shock wave generator, and the selectable energy flow density range is 0.03–0.5 mJ/mm2. The anesthetized rabbit lied on its side on the treatment table, and the experimental site hair was cut off. Then, coupling gel was applied between the skin and the water bladder to reduce energy loss. Then, the left femoral condyles of the grouped rabbits were subjected to ESW treatment every 3 days for 4 weeks with two energy flow density of 0.12 mJ/mm^2^ or 0.5 mJ/mm^2^, pulse 2,000 times, frequency 4 Hz, focal length (penetration depth) 10 mm, and no treatment was done on the right side as a self-control. The rabbits were sacrificed at 4 weeks after the operation.

### Micro-CT Analysis

We scanned femur samples dissected from rabbits and analyzed them through micro-CT (Quantum GX, PE). Micro-CT scans were performed under the same conditions: voltage 80 kV, current 80 μA, spatial resolution 14 μm, scanning 500 continuous sections. Three-dimensional reconstruction was performed on each specimen, and then the data was collected and analyzed automatically through computer software to analyze the number of trabecular bones (Tb.N), trabecular bone thickness (Tb.Th), trabecular bone space (Tb.Sp), bone volume fraction (BV/TV), and other indicators.

### MTT Assays *In Vitro*

BMSCs treated with ESW therapy were cultured in 48-well plates at a density of 5,000 cells/cm^2^. We added 40 μl of MTT to each well at the corresponding time point for 4 h, and then discarded the liquid and added 150 μl of DMSO, shake for 10 mins. OD value of 495 nm wavelength was detected in the enzyme-linked immunoassay instrument.

### Immunofluorescence Staining

To examine dynamic bone formation, we subcutaneously injected 0.5% tetracycline (Sigma-Aldrich, 25 mg/kg body weight) in PBS into rabbits 14 days and 0.1% calcein (Sigma-Aldrich, 20 mg/kg body weight) in PBS into rabbits 3 days before sacrifice. After execution, the bones of bilateral femoral condyles were taken and fixed with 10% neutral formaldehyde without decalcification. After dehydration, they are immersed in plastic liquid one by one, embedded in plastic for 3 weeks, and solidified into a block. Use a rotary hard tissue microtome to cut into slices with a thickness of 150 microns. Use a fluorescence microscope to observe the fluorescent double labeling of new bone trabeculae.

### Ponceau Staining

We prepared femur samples dissected from rabbit into paraffin sections and deparaffinized them. Then, we used chromium treatment and washed them with tap water for three times, and stained them with Weigert hematoxylin for 5–10 min, and then, washed thoroughly with distilled water and stained with Masson Ponceau acid complex red solution for 5–10 min, and then soaked in 2% glacial acetic acid for a while. Without washing, the sections were dyed directly with aniline blue for 5 min and soaked in 2% glacial acetic acid for a while.

### Three-Point Bending Experiment

We used mechanical-testing machine equipped with a 500 NM-SI sensor (Celtron Technologies Inc.) to run three-point bending test. Two-end-support points and one-central-loading point were set for the three-point bending test. The length span between two-support-points was 60% of the total bone length. Each bone was loaded at a constant speed of 0.160 mms^−1^ until failure. We collected biomechanical measurement data from the load-deformation curves. The maximum load (N) and stiffness (N/mm) were recorded.

### Western Blot Analysis

Western blotting was performed as previously described (8). We used M-PER Protein Extraction Reagent (CST) supplemented with a protease inhibitor cocktail (CST) to lyse cells. Then, we used a BCA assay (CST) to measure protein concentrations and normalized to the extraction reagent. Equal amounts of the extracts were loaded and subjected to SDS-PAGE, transferred onto nitrocellulose membranes, and then blotted as reported. Antibodies specific to rabbits GADPH (Abcam), SMAD2 (Abcam), and secondary antibodies were purchased from Abcam.

### Statistical Analysis

All data are presented as the mean ± standard deviation (S.D.). Two groups were assessed by Student’s t-test and multiple groups were compared using one-way analysis of variance (ANOVA). Differences at P < 0.05 were considered significant.

## Results

### ESW Therapy Promoted Osteoblast Differentiation *In Vitro*

**Figure 1:**
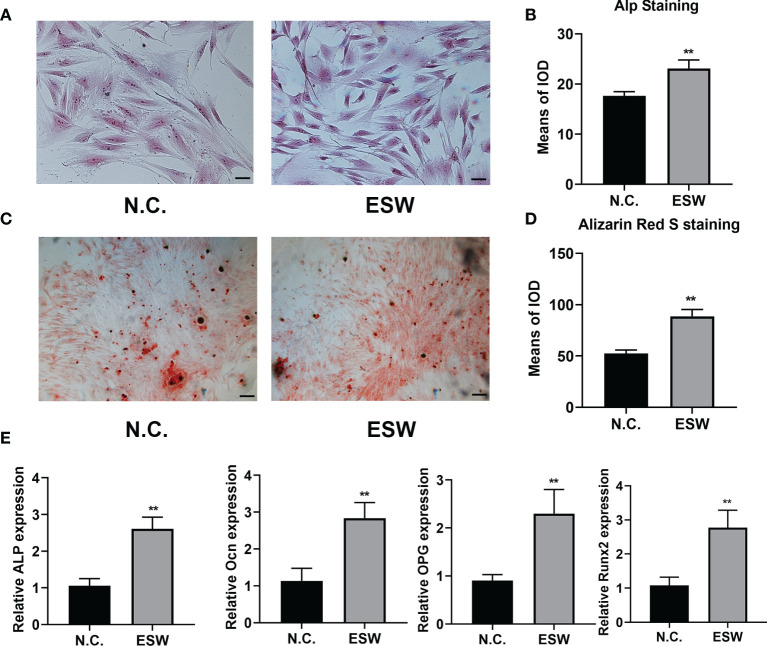
ESW therapy promoted osteoblast differentiation *in vitro*. **(A)** The ALP staining of BMSCs cultured in DMEM treated with N.C. or ESW, and quantitative analysis, Scale bar: 100 μm **(B)**; **(C)** The Alizarin Red S staining of BMSCs cultured in DMEM treated with N.C. or ESW, and quantitative analysis, Scale bar: 100 μm **(D); (E)** The mRNA expression of ALP, OCN, OPG, and RUNX2 of BMSCs cultured in a-MEM treated with N.C. or ESW after 24 h. Data are reported as the mean ± SD. **p < 0.01.

In order to determine the effect of ESW therapy on the osteogenic differentiation of BMSCs cells, we performed alp staining and found that ESW therapy enhanced alp staining at day 7 significantly (**Figures 1A, B**). Furthermore, we found that increased mineral deposition in BMSCs treated with ESW compared with its negative control (**Figures 1C, D**). In addition, compared with negative control, ESW therapy significantly increased expressions of osteogenesis related markers of ALP, Ocn, osteoprotegerin (OPG), and the runt-related transcription factor 2 (Runx2) (**Figure 1E**). Consistent with the results of BMSCs culture of RAW264.7 cells showed that osteoclast differentiation was inhibited by ESW therapy (**Figure S2**). Thus, these data suggest that osteogenesis was promoted by ESW therapy.

### ESW Treatment Increased Bone Formation *In Vivo*

**Figure 2:**
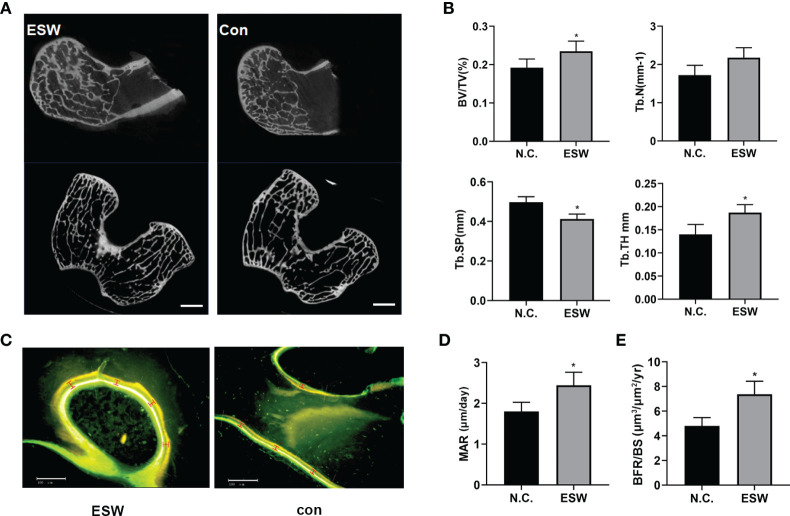
ESW treatment increased bone formation *in vivo*. **(A)** Representative micro-CT images in femur from rabbits treated with ESW or Con. Scale bar: 500 μm. **(B)** Quantitative micro-CT analysis of femur from rabbits treated with ESW or Con. **(C)** Representative images of calcein (Green) and tetracycline (yellow) labeling of trabecular bone from rabbits treated with ESW or Con. Scale bar: 500 μm. **(D)** the mineral apposition rate (MAR) and **(E)** the quantification of the BFR per bone surface (BFR/BS) of the femur from rabbits treated with ESW or Con. (n = 7, per group). Data are reported as the mean ± SD. *p < 0.05.

To investigate the role of ESW therapy *in vivo*, we performed ESW treatment on rabbit femoral condyles. Micro-CT showed that increased trabecular bone volume, number, and thickness, and decreased trabecular separation in rabbit femurs compared with those controls (**Figures 2A, B**, **S1**). Calcein (Green) and tetracycline (yellow) double labeling indicated that ESW therapy group had significantly increased mineral apposition rates (MAR) and the quantification of the BFR per bone surface compared control group (**Figures 2C–E**). Therefore, all of these results indicated that ESW therapy promoted osteoblast development *in vivo*.

### High Density or Frequency of Energy Inhibited Osteogenesis

**Figure 3:**
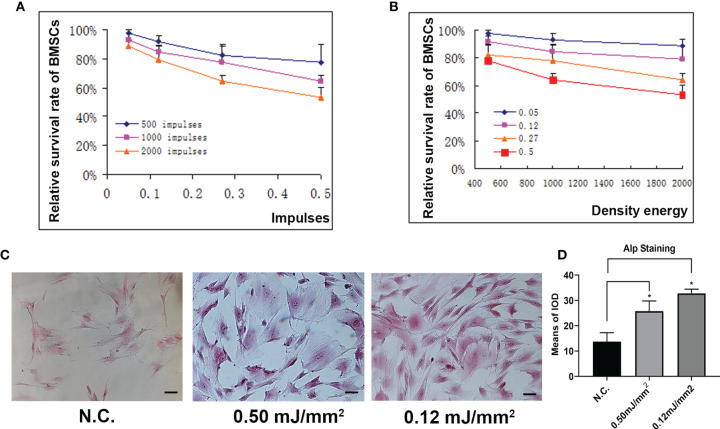
Different density or frequency of energy had little effects on osteogenesis. **(A)** Relative survival rate of BMSCs treated with ESW at different impulses. **(B)** Relative survival rate of BMSCs treated with ESW at different density of energy. **(C)** Representative images of ALP staining in BMSCs treated with ESW at different density of energy and quantitative analysis **(D)** Scale bar: 100 μm. Data are reported as the mean ± SD. *p < 0.05.

To determine the effects of different energy density and impulse times on osteoblast differentiation. We applied different energy density and impulse times on BMSCs and found that the survival rate of BMSCs was inhibited as the energy flow density or the number of impulses increased (**Figures 3A, B**). Furthermore, alp staining showed that low energy flow density enhanced osteoblast differentiation compared to those treated with high energy flow density (**Figures 3C, D**). In total, these data reported that osteoblast development was inhibited as the energy flow density or the number of impulses increased.

### Administration of ESW Can Be a Potential Therapy of Osteoporosis

**Figure 4:**
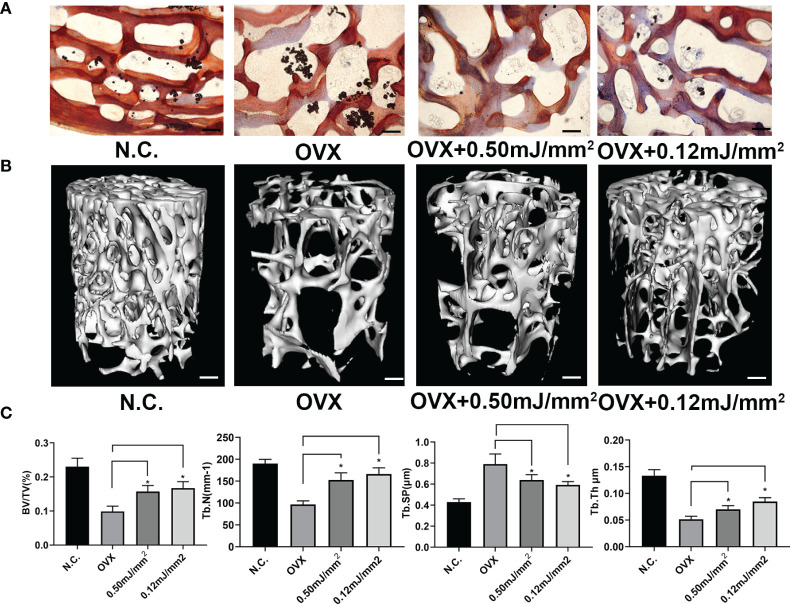
Administration of ESW can be a potential therapy of osteoporosis. **(A)** Representative images of Ponceau Staining in Rabbit femurs. Scale bars =500 μm. **(B)** Representative micro-CT images in femur from rabbits. Scale bars =500 μm. **(C)** Quantitative micro-CT analysis of femur from rabbits. Data are reported as the mean ± SD. *p < 0.05.

To investigate the therapeutic potential of ESW therapy on age-related osteoporosis. We generated rabbit osteoporosis model as described previously. Osteoporosis rabbits treated with 0.12 mJ/mm^2^ or 0.5 mJ/mm^2^ showed increased new bone trabeculae by Ponceau Staining (**Figure 4A**). Furthermore, micro-CT showed Osteoporosis rabbits treated with 0.12 mJ/mm^2^ or 0.5 mJ/mm^2^ showed increased trabecular bone volume, number, and thickness, and decreased trabecular separation relative to OVX groups (**Figures 4B, C**). Previously, SMAD2 has been reported to associate with osteogenesis and promote bone formation through TGF-β/SMAD2 pathway. In this study, we found that ESW can promote endogenous levels of SMAD2 protein expression (**Figure S3**). In addition, we found that Osteoporosis rabbits treated with 0.12 mJ/mm^2^ showed increased trabecular bone volume, number, and thickness, and decreased trabecular separation relative to Osteoporosis rabbits treated with 0.50 mJ/mm^2^, which were consistent with Western Blot analysis. In conclusion, these results show that ESW therapy promoted bone formation and prevented bone loss on osteoporosis through TGF-β/SMAD2 pathway and osteoblast differentiation can be more effectively promoted by ESW therapy with low energy flow density.

## Discussion

Skeleton is a dynamic organ which undergoes a constant remodeling process mediated by osteoclasts and osteoblasts. Osteoporosis is a systemic bone loss disorder resulting from a negative remodeling balance when bone destruction by osteoclasts exceeds bone formation by osteoblasts (9). Pharmacotherapy is the primary method for the treatment of osteoporosis *via* application of Antiresorptive drugs (bisphosphonates, denosumab) or Anabolic drugs (teriparatide) (10). However, various side effects and gradually decreased efficacy have limited the utilization of these agents (11, 12), necessitating the development of safer and effective therapies for the management of osteoporosis.

ESWT was firstly applied in clinical medicine around the 1980s to disintegrate kidney stones (13). With features of non-invasion and pro-regeneration, ESWT has been introduced in trauma and orthopedic discipline for the treatment of various musculoskeletal diseases (14–16). It was reported that ESWT induced new bone formation in rabbits (3). Clinical study showed that a single session of ESWT significantly increased local bone mineral density (BMD) in postmenopausal osteoporotic patients without obvious complications (17). In accord with the previous studies, we demonstrated that both low- and high-intensity ESWT stimulated local new bone formation and improved micro-architecture in osteoporosis rabbit models, and the efficacy lasted at least 6 months. Nevertheless, we only tested the site of distal femur. The effectiveness and safety for other positions, such as vertebrae where spinal cord situates, need further examination. In addition, the localized efficacy of ESWT may limit the application of shock wave therapy for systemic bone disorders. Different parameters of ESWT (frequency, focal distance, focused, or radial) should be investigated in future.

The SMADs family are BMP signaling proteins, in which SMAD2 can transform TGF-β pathway signals, thereby inducing the expression of osteoblast-related genes and promoting differentiation of osteoblasts (18, 19). In our study, we found ESW therapy promoted bone formation and prevented bone loss on osteoporosis. Furthermore, levels of SMAD2 protein expression could be up-regulated by ESW therapy. Thus, we believe that ESW could promote osteoblast differentiation through the TGF-β/SMAD2 pathway and further stimulate bone formation.

Stress stimulation plays an important regulatory role in bone growth, absorption, and reconstruction. As a physical stimulus, ESW produces tensile and compressive stress on the surface of tissue cells when conducting between different media, and the cells convert mechanical signals into biochemical signals in respond to mechanical impact (20, 21). In this study, we found that low-intensity ESW (0.12 mJ/mm2) promoted the proliferation and osteogenic differentiation of BMSC *in vitro*, while high-intensity ESW (0.5 mJ/mm2) suppressed BMSC proliferation. However, limited by current situation, we have not explored the effects of ESW on osteoclastic cell line, which may provide insights into better understanding of the ESW effects on skeletal system. We cannot exclude the possibility that other mechanism of ESWT-mediated increase in bone formation may exist, which warrants further investigation (22).

In sum, the present study demonstrated that ESWT promoted local new bone formation, enhanced the quantity and quality of local cancellous bone in osteoporosis rabbit models. The positive effects of ESWT on proliferation and osteogenic differentiation of BMSC may be an important mechanism. These results could provide theoretical basis for the prevention and treatment of bone metabolic diseases.

## Data Availability Statement

The raw data supporting the conclusions of this article will be made available by the authors, without undue reservation.

## Ethics Statement

The animal study was reviewed and approved by the Animal Care and Use Committee of Fourth Military Medical University.

## Author contributions

BL and RW contributed to the conception and design of this study, the performance of experiments, interpretation, data analysis, and manuscript writing. XH, YO, ZJ, and SL performed data analysis and interpretation. YZ, HX, and BC contributed to the design of this study, acquiring financial support, data analysis, interpretation, manuscript writing, and the final approval of the manuscript. All authors contributed to the article and approved the submitted version.

## Funding

This study was supported by the National Natural Science Foundation of China (No. 81972895, 81000819).

## Conflict of Interest

The authors declare that the research was conducted in the absence of any commercial or financial relationships that could be construed as a potential conflict of interest.
