# Supplementary material for: Extracorporeal Shock Wave Therapy Promotes Osteogenic Differentiation in a Rabbit Osteoporosis Model
Source: Front Endocrinol (Lausanne). 2021 Mar 25;12:627718. doi: 10.3389/fendo.2021.627718 (PMC8027252; doi:10.3389/fendo.2021.627718)
Supplement: Supplementary file 1 [file DataSheet_1.docx]

**supplementary material**


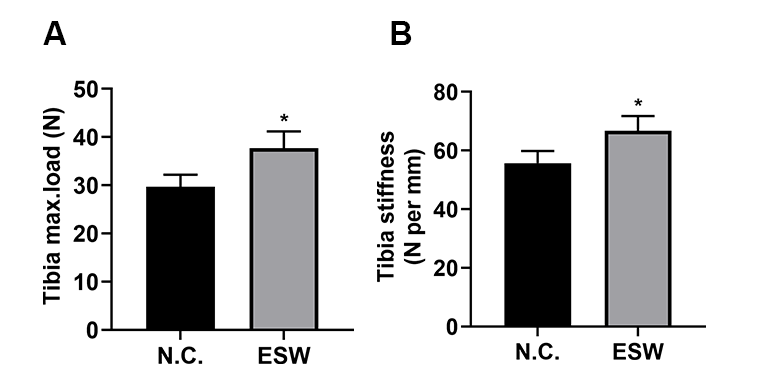


**Figure S1** **ESW treatment increased bone formation in vivo.** (A, B) Three-point bending measurement of tibia maximum load. (n=7). Data shown as mean±s.d. * P<0.05


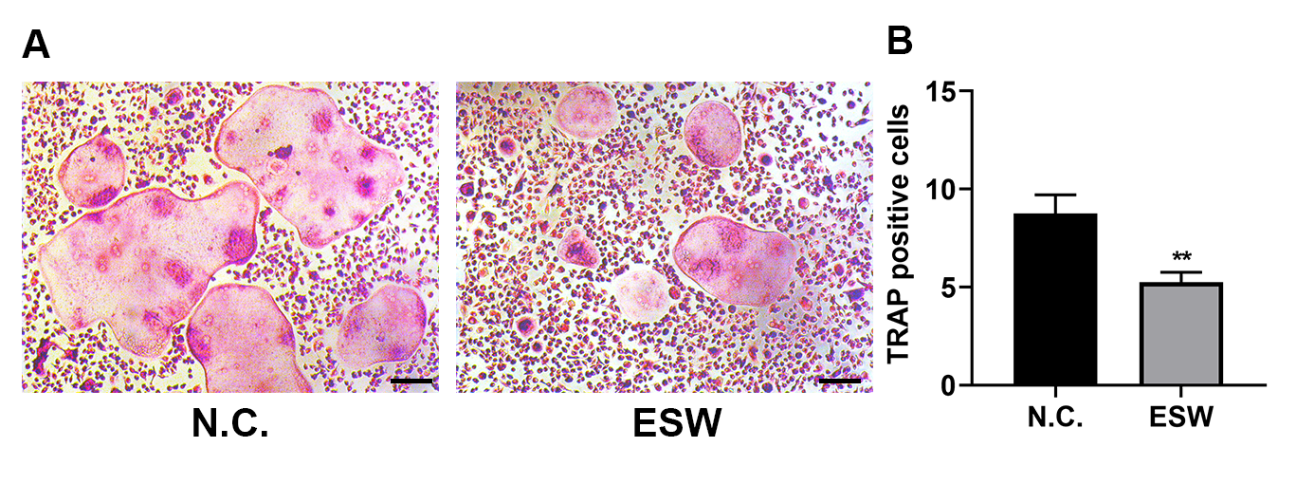


**Figure S2** **ESW therapy inhibited osteoclast differentiation in vitro. (A)** The TRAP staining of RAW264.7 cultured in a-MEM treated with N.C. or ESW , and (B) quantitative analysis, Scale bar: 100μm


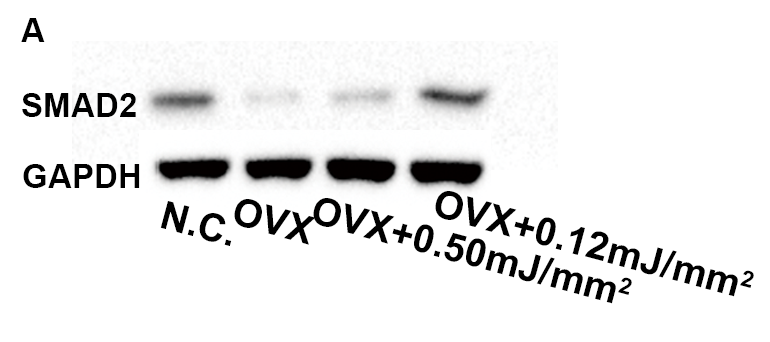


**Figure S3 ESW therapy might activated** **TGF pathway.** (A) Western blot analysis of SMAD2.
